# An Assay to Detect *In Vivo* Y Chromosome Loss in *Drosophila* Wing Disc Cells

**DOI:** 10.1534/g3.112.002899

**Published:** 2012-09-01

**Authors:** Janos Szabad, Hugo J. Bellen, Koen J. T. Venken

**Affiliations:** * Department of Biology, University of Szeged, H-6720 Szeged, Hungary, and; † Department of Molecular and Human Genetics; ‡ Howard Hughes Medical Institute, and; § Program in Developmental Biology, Baylor College of Medicine and Neurological Research Institute, Houston, Texas 77030

**Keywords:** chromosome loss, Y chromosome, multiple wing hair, wing mosaic spots, *Drosophila*

## Abstract

Loss of the *Y* chromosome in *Drosophila* has no impact on cell viability and therefore allows us to assay the impact of environmental agents and genetic alterations on chromosomal loss. To detect *in vivo* chromosome loss in cells of the developing *Drosophila* wing primordia, we first engineered a *Y* chromosome with an *attP* docking site. By making use of the ΦC31 integrase system, we site-specifically integrated a genomic transgene encompassing the *multiple wing hair* (*mwh*) locus into this *attP* site, leading to a *mwh*^+^*Y* chromosome. This chromosome fully rescues the *mwh* mutant phenotype, an excellent recessive wing cell marker mutation. Loss of this *mwh*^+^*Y* chromosome in wing primordial cells then leads to manifestation of the *mwh* mutant phenotype in *mwh*-homozygous cells. The forming *mwh* clones permit us to quantify the effect of agents and genetic alterations by assaying frequency and size of the *mwh* mosaic spots. To illustrate the use of the *mwh*^+^*Y* loss system, the effects of four known mutagens (X-rays, colchicine, ethyl methanesulfonate, and formaldehyde) and two genetic conditions (loss- and gain-of-function *lodestar* mutant alleles) are documented. The procedure is simple, sensitive, and inexpensive.

Elaborated mechanisms ensure the maintenance of genome integrity and stability in cells (Musacchio and Salmon 2007; Vakifahmetoglu *et al.* 2008). Loss of a chromosome usually disrupts the genetic balance, and the ensuing condition leads mostly to cell death. However, some monosomic cells can occasionally survive and propagate their unusual condition to their descending cells. The abnormal conditions may lead to human disabilities such as mental disabilities, miscarriage, and cancer. In humans, aneuploidy, which includes monosomy, has been regarded as a hallmark of cancer (Pellman 2007; Torres *et al.* 2008; Williams *et al.* 2008; Li *et al.* 2010; Tang *et al.* 2011).

Changes in the cell’s heritable material can be classified into three major types: (1) point mutations, (2) chromosomal breaks that may alter the amount of DNA in the cells, and (3) changes in chromosome number. There have been quite a number of assays developed to detect the first and the second types of mutations, and several of those have been used on a large scale (Zeiger 2004; Claxton *et al.* 2010). To detect gain and/or loss of the chromosomes, a number of the so-called aneuploidy test procedures were elaborated mostly in the 1980s and 1990s. They are proficiently overviewed in panel reports such as the Food and Drug Administration’s Redbook or the OECD Test Guidelines for Genotoxicity and Mutagenicity Testing. The aneuploidy test procedures usually make use of yeasts, *Drosophila*, or cultured mammalian cells. There are two main reasons why they are not routinely used and included in the batteries of mutagenicity test procedures. (1) The aneuploidy-detecting assays are not sensitive enough to observe rare events in a generally limited number of target cells. The high background noise, especially in the karyotyping-based procedures, sets a strong limit on the use of several of the proposed procedures. (2) Most of the aneuploidy test procedures are quite sophisticated and are usually rather expensive.

To overcome these issues, we developed an assay to detect *in vivo* loss of the *Y* chromosome in cells of the developing wing imaginal discs of *Drosophila melanogaster*. We selected to develop this assay based on the following observations and data. (1) Gain and/or loss of the *Y* chromosome with its nine Y-linked genes (Carvalho *et al.* 2001) has no impact on viability of the diploid imaginal disc cells. (2) Importantly, several thousand cells can be exposed to physical, chemical, or biological “treatments” in a single developing wing disc. Roughly one-half of the proliferating wing disc cells will give rise to the wing blade, a chitinous structure that is easy to mount and analyze. By using an appropriate marker, the individual genotype of about 30,000 wing blade cells can be determined.

In this report we (1) describe the generation of a *Y-attP* chromosome that permits site-specific integration of transgenes using the ΦC31 integrase, (2) the insertion of an *multiple wing hair* (*mwh*^+^) transgene into the Y chromosome, and (3) document the use of the *mwh*^+^*Y* chromosome to detect and quantitatively characterize the *in vivo* loss of the *mwh*^+^*Y* chromosome by quantifying the effects of X-rays, colchicine, ethyl methanesulfonate (EMS), and formaldehyde as well as the loss- and the gain-of-function *lodestar* mutant alleles. The assay is simple, sensitive, takes approximately 1 week to complete, and is very inexpensive.

## Materials and Methods

### Construction of the *mwh*^+^Y chromosome

To generate an *attP* docking site in the Y chromosome, we made use of the *P* conversion or replacement method (Sepp and Auld 1999). The donor strain was *y w^67c23^*; *P{y^+t7.7^ = CaryP}attP1* integrated into chromosome 2R (Groth *et al.* 2004; Markstein *et al.* 2008). The acceptor strain was y w^*^/Dp(2;Y)G, *P{w^+mC^ = hs-hid}Y* with a segment of the second chromosome integrated into the *Y* chromosome (Starz-Gaiano *et al.* 2001). Mobilization of *P{y^+t7.7^ = CaryP}attP1* was done using y w^*^; *L/CyO*; *D/TM3*, *ry^RK^ Sb P{Delta2-3}99B* as a transposase source (Robertson *et al.* 1988). Presumable mobilization events to the *Y* chromosome were identified as *yellow^+^* marked males that maintained the *L* or *CyO* chromosomes, indicative for loss of the original donor chromosome. Subsequently, linkage of the *yellow^+^* marker, associated with the *attP* site, to the *Y* chromosome was verified after simple chromosome segregation of the *Dp(2;Y)G*, *P{y^+t7.7^ = CaryP}attP Y* chromosome (from now on abbreviated as *Y-attP*) from males in the parental generation to males of the next generation. Several independent *Y-attP* chromosomes were generated using this method. To ensure the loss of the *hs-hid* portion contained within the original *P{w^+mC^ = hs-hid}* element, fertilized females were allowed to lay eggs for 3 days, followed by the removal of the adults, further development of the larvae for 2 more days, and a heat shock of the larvae at 37° for 1 hr. After eclosion of the developing pupae, no males where observed in the *P{w^+mC^ = hs-hid}* element containing stock (0 XY males, 0%; 0 X0 males, 0%; 440 XX females, 100%; 0 XXY females, 0%), whereas males did eclose from the *Y-attP* stock, indicating full removal of the *P{w^+mC^ = hs-hid}* element (70 XY males, 31.1%; 1 X0 males, 0.4%; 154 XX females, 68.5%; 0 XXY females, 0%).

To test the receptiveness of the *Y-attP* chromosomes, we tested the integration of a *white*^+^ containing *attB-P[acman]-Ap^R^* clone by coinjection with the ΦC31 integrase encoding mRNA, as described earlier (Groth *et al.* 2004; Venken *et al.* 2006). Stocks were then generated that contain the *Y-attP* chromosome as well as the ΦC31 integrase present in y M{vas-int.B}ZH-2A w^*^ on the X-chromosome (Bischof *et al.* 2007). These stocks were then retested for receptiveness using *attP-P[acman]-Ap^R^* (Venken *et al.* 2006).

The entire *mwh* locus is present in the FlyFos-030330 clone: DNA clones from the original fosmid library are marked with an eye-expressed DsRed fluorescent marker driven by the *3xP3* eye specific promoter, for which transgenic flies can easily be identified in a *white* mutant background (Ejsmont *et al.* 2009). Moreover, these clones do not contain insulator sequences shielding the transgene from the surrounding environment; however, that ended up not being a problem, as exemplified in the *Results* section. The FlyFos-030330 sequence was integrated into the *Y-attP* chromosome as well as into the VK16 docking site in the 47C cytological region of the second chromosome (Venken *et al.* 2006, 2009), resulting in *Dp(2;Y)G*, *P{y^+t7.7^ = CaryP FlyFos-030330}attP Y* (from now on abbreviated as *mwh*^+^*Y*) and PBac{y^+^-attP-3B *FlyFos-030330*}VK00016 (from now on abbreviated as VK16 *mwh*^+^). Hence, the engineered *mwh*^+^*Y* chromosome (as well as the VK16 *mwh*^+^ line) carries the following markers: *yellow^+^*, DsRed, and *mwh*^+^. In this stock, X0 males appeared at a very low frequency (3/1366 males, 0.2%, that were nonrescued *mwh* mutant and sterile).

### The *w*/*mwh*^+^*Y*; *mwh* strain

The *mwh*^+^*Y* chromosome was then integrated into a *w^1118^*; *mwh* (*mwh*^1^) background to create a *w^1118^* / *mwh*^+^*Y*; *mwh* stock. The *w^1118^* (shortly *w*) allele allows the detection of the fluorescent DsRed marker (that marks the presence of the *mwh*^+^*Y* chromosome) and hence the convenient identification of the occasional loss of the *mwh*^+^*Y* chromosome in *w*/0; *mwh* males.

**Figure 1:**
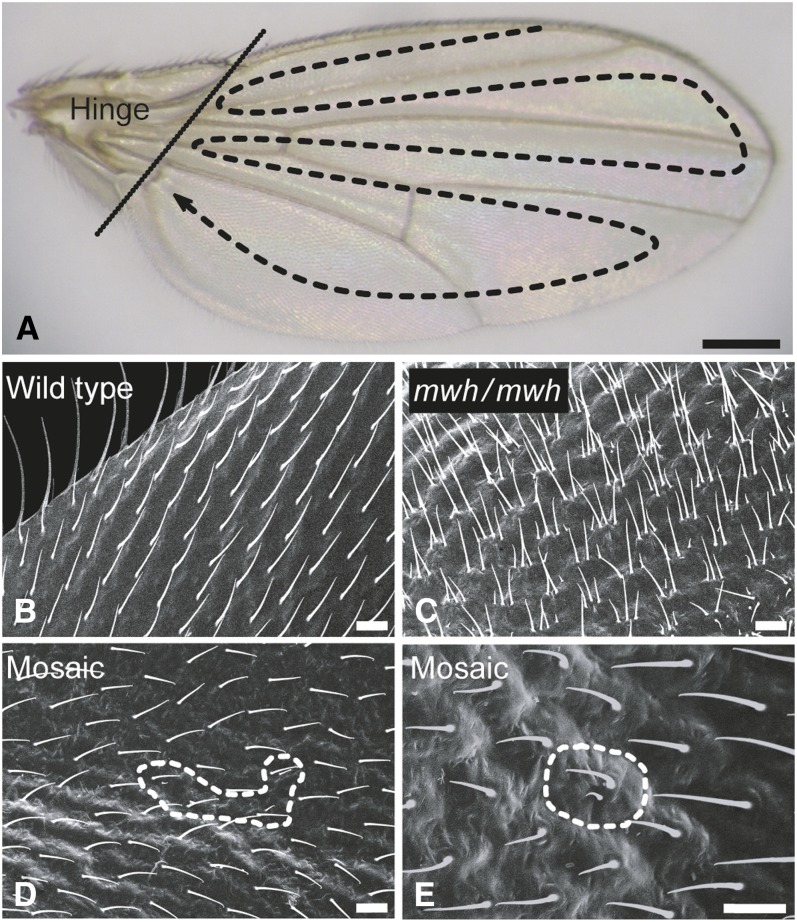
Wings and wing hairs. (A) *Drosophila* wing with the screening path (dashed arrow) drawn onto the blade. The hinge region (left of stripe) was omitted from the clone screen. (B,C) Scanning electron microscope photographs of wing hairs from a wild-type (B) and from an *mwh* (C) homozygous wing. (D,E) Two *mwh* mosaic spots (encircled by dashed lines). One is composed from four (D) and the other from a single (E) *mwh* homozygous cell. Scale bar for A, 200 μm. Scale bar for B−E, 10 μm.

Wing blade cells homozygous for the *mwh* marker mutation, linked to the third chromosome, produce two to five trichomes per cell instead of the regular single trichome seen in wild-type or *mwh*/*mwh*^+^ heterozygous cells (Figure 1). The trichomes (hairs) are usually short and possess abnormal polarity (Yan *et al.* 2008). Single *mwh* homozygous cells can easily be detected in the midst of broad fields of wild-type cells (Szabad *et al.* 1983).

### Treatments

Eggs were collected in 8-hr shifts from the *w*/*mwh*^+^*Y*; *mwh* strain on standard *Drosophila* corn meal food with live yeast and kept at 25° throughout. We also raised flies on the Formula 4-24 Instant *Drosophila* Medium (Carolina Biological Supply Company) to assess if the culture conditions can influence the frequency of *mwh*^+^*Y* chromosome loss. The hatching larvae developed on standard *Drosophila* food and were treated 84−92 hr after egg laying, the event that marks the commencement of embryogenesis. There are approximately 5000 to 6000 cells in a developing wing blade primordium at this mid-third instar stage of development (Bryant and Levinson 1985). The remaining 28−36 hr to pupariation (at 120 hr after egg laying) allow approximately three rounds of mitoses before the cessation of cell proliferation (Bryant and Levinson 1985). The 84- to 92-hr-old larvae keep on foraging for 4−12 more hours (Rodriguez Moncalvo and Campos 2009). Hence, the tested chemicals can enter their digestive tract and reach the cells of the wing primordia.

For treatments, the 84- to 92-hr-old larvae are floated off the food with a 14% NaCl solution, collected on a nylon mesh, washed with tap water, dried up briefly, and transferred onto standard *Drosophila* food into which the substance to be tested was mixed. Larvae finished development on this food in case of colchicine and formaldehyde treatments (Table 1). The floated larvae can also be immersed into a solution for exposure to chemicals, as was the case during EMS treatment, or irradiated before transferring back to standard *Drosophila* food (Table 1).

**Table 1:** Features of *mwh* mosaicism

Treatment and/or Genotype*^a^*	Wing, *N*	*mwh* Clone, *n*	*mwh* Clone Frequency, *n*/*N*	Size Class (I-VIII)*^b^* and the Number of *mwh* Cells per Clone								Average Clone Size*^c^* (*mwh* Cell per Clone), *m*	Frequency of Clone Induction, *f*
				I. 1	II. 2	III. 3−4	IV. 5−8	V. 9−16	VI. 17−32	VII. 33−64	VIII. 65−128		
Control	108	164	1.5	96	39	22	5	2	0	0	0	1.7 ± 1.0	1.7 × 10^−4^
*Su-var(2)1^03^*	40	56	1.4	27	17	7	4	1	0	0	0	1.9 ± 1.0	1.8 × 10^−4^
*Su(var)3-9^ptn^*	32	52	1.6	28	13	9	2	0	0	0	0	1.8 ± 0.9	1.9 × 10^−4^
X-rays; 1000 Rad (150 kV, 0.5 mm Al; 500 Rad/min)	12	119	9.9**	37	31	19	15	9	4	2	2	2.6 ± 1.5	17.2 × 10^−4^
X-rays; *mwh*^+^ *VK16*; 1000 Rad (150 kV, 0.5 mm Al; 500 Rad/min)	40	7	0.2**	5	1	1	0	0	0	0	0	1.4 ± 0.9	0.2 × 10^−4^
Colchicine (1 μg/mL in the food. From 84-92 hAEL on)	4	63	15.8**	8	15	16	11	6	7	0	0	2.8 ± 1.5	29.4 × 10^−4^
EMS (25 mM for 4 hr at 84−92 hAEL)	18	57	3.2*	24	16	9	2	4	2	0	0	2.2 ± 1.4	4.6 × 10^−4^
Formaldehyde*^d^* (0.05M in the food. from 84−92 hAEL on)	46	136	2.9**	92	31	12	1	0	0	0	0	1.4 ± 0.7	2.8 × 10^−4^
*lds^Hor-D^*/*lds*^+^	14	73	5.2**	38	26	7	2	0	0	0	0	1.7 ± 1.0	5.9 × 10^−4^
*nub-Gal4*; *UAS-lds^Hor-D^*; *lds*^+^/*lds*^+^	24	247	10.3^**^	99	64	45	31	6	2	0	0	2.2 ± 1.2	15.1 × 10^−4^
*lds^hor-rvP2l^*/*Df(3R)ED5218*	24	164	6.8^**^	84	43	23	11	3	0	0	0	1.9 ± 1.0	8.7 × 10^−4^

* and ^**^ indicate significantly different from the control at *P* < 0.05 and *P* < 0.01, respectively. hAEL, hours after egg laying.

a All the males carried the *w^1118^*-labeled X, the *mwh*^+^*Y* chromosome, and were homozygous for *mwh*. *Su-var(2)1^03^* is a dominant suppressor mutation of position-effect-variegation and *Su(var)3-9^ptn^* is an exceptionally strong position-effect-variegation enhancer mutation (Schotta *et al.* 2003; Ebert *et al.* 2004).

b The minimum number of cell divisions (I−VIII) required—after the loss of the *mwh^+^Y* chromosome—for the formation of clones composed from 1, 2, 3−4, etc., *mwh* cells. It was assumed that only one of the daughter cells becomes *mwh*-labeled after the loss of the *mwh*^+^*Y* chromosome during mitosis.

c Calculated from the average size class by making use of the linear relationship between size classes (I−VIII) and the log average clone size within the different size classes.

d 0.05M formaldehyde mixed into the food allows 50% of the larvae develop to adult (Szabad *et al.* 1983).

To assess the “genetic effects” on loss of the *mwh*^+^*Y* chromosome, we analyzed wings of (1) *w*/*mwh*^+^*Y*; *mwh lds^hor-rvP2^*/*mwh Df(3R)ED5218* males that lack the product of *lodestar* (*lds*), a member of the Snf2 family of helicase-related genes (Szalontai *et al.* 2009). The abbreviations are as follows: *lds^hor-rvP2^* is a complete loss-of-function *lds* allele (Szalontai *et al.* 2009), and *Df(3R)ED5218* is a small deficiency generated by the deletion of the segment encompassed between two *FRT* site containing transposons (Ryder *et al.* 2007) that removes *lds* and a few adjacent loci. (2) The *w*/*mwh*^+^*Y*; *mwh lds^Hor-D^*/*mwh lds*^+^ males carried *lds^Hor-D^*, a dominant, chromosome instability causing mutation in *Drosophila* (Szabad *et al.* 1995; Szalontai *et al.* 2009). (3) In the *w*/*mwh*^+^*Y*; *nub-Gal4*/*UAS-lds^Hor-D^*-CFP; *mwh* males the *nub-Gal4* driver (Calleja *et al.* 2000) ensures expression of a *UAS-lds^Hor-D^*-CFP transgene (inserted into the second chromosome) in wing imaginal disc cells in an *mwh* homozygous background.

### Wing preparation and scoring

The *w*/*mwh*^+^*Y*; *mwh* male flies were aged for 1 to 2 days after eclosion. They were dipped first into a 96% ethanol for a few seconds, transferred into water, and their wings were detached. The wings were mounted in Faure’s mounting medium such that wings of every male were positioned in pair-wise fashion. Except for the hinge region, the wings were screened at ×400 magnification (Figure 1). The number and size of the *mwh* clones were recorded. The screened area of a wing blade contains about 30,000 cells (Garcia-Bellido and Merriam 1971). In determining the number of *mwh* clones and their size, we followed a published protocol (Graf *et al.* 1984). Classification of the clones composed of ≥3 *mwh* homozygous cells is straightforward. Single cells were considered to be *mwh* homozygous if they carried at least two trichomes that pointed into different directions. Two *mwh* cells were classified as a single clone if they were on the same wing surface and were not separated by more than three normal cells. To determine the average clone size, the *mwh* clones were classified into size classes that represent the minimum number of cell divisions required, following the loss of the *mwh^+^Y* chromosome, for the formation of clones composed from 1, 2, 3−4, 5−8, etc. *mwh* cells (Table 1). We presumed that the *mwh*^+^*Y* chromosome is lost from only one of the daughter cells during mitosis.

## Results

### Constructing the *mwh^+^Y* chromosome

**Figure 2:**
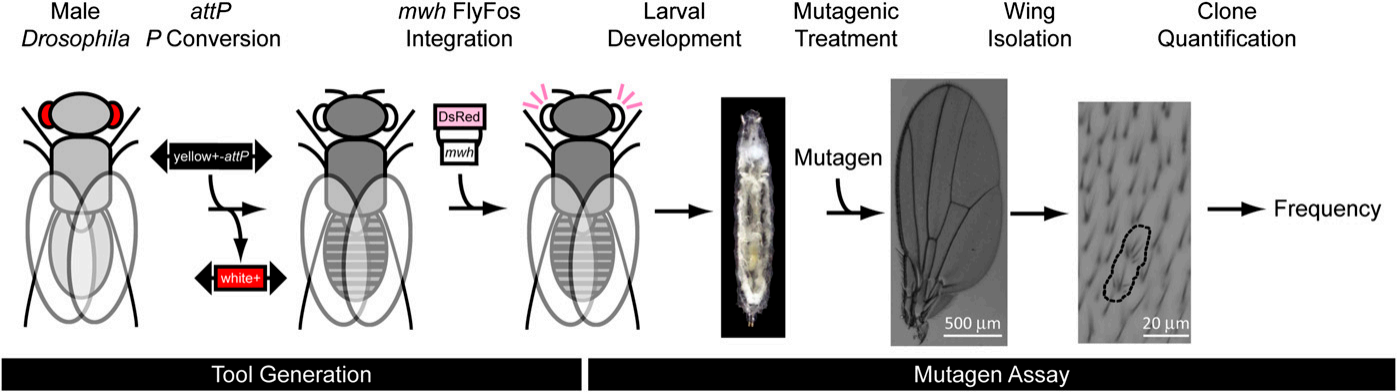
Overview of the strategy. Tool-generation phase: *P* element conversion was used to replace a *white^+^* marked *P* element with an *attP* site and *yellow^+^* marker containing *P* element. A FlyFos clone containing the entire *mwh* locus was integrated into the *attP* site using the ΦC31 integrase. This transgene was combined into an *mwh* mutant background and rescued the homozygous *mwh* phenotype. Mutagen assay phase: The resulting flies were allowed to develop until a late larval stage and treated with mutagens (see *Materials and Methods*). Adult wings were removed, analyzed for the presence of *mwh* clones, and the frequency of such clones calculated.

To screen for chromosome loss, we generated a *mwh*^+^*Y* chromosome into which an *mwh****^+^*** genomic rescue fragment is integrated at an *attP* docking site (see *Materials and Methods*). The *mwh*^+^ transgene in this chromosome rescues the *mwh* mutant phenotype. The resulting *w/mwh*^+^*Y*; *mwh* stock was used to detect *in vivo* loss of the *mwh*^+^*Y* chromosome (Figure 2). Note that the presence of an efficient *attP* docking site on the *Y* chromosome also can be used to integrate other markers that may allow optimal live labeling of male embryos and young larvae.

Loss of the *mwh*^+^*Y* chromosome during mitosis leads to the formation of a cell without *mwh*^+^ function. This cell is fully viable and propagates its new character onto its descending cells during the oncoming mitoses. The daughter cells will stay together and form a *mwh* clone (mosaic spot), in an *mwh* homozygous genetic background, after metamorphosis in the wing blade. In other words, groups of cells that display the *mwh* mutant phenotype on the wings of *w/mwh+Y*; *mwh* males should reflect events involving the loss of the *mwh+Y* chromosome.

### The background *mwh* clone frequency

Principles of the quantification are as follows. Assuming equal contribution of the wing disc cells to the wing blade, the number of cells in a wing disc primordium at the time of *mwh* clone induction is *C*/2*m*, where *C* is the number of the screened cells in a wing blade [*C* = 30,000 (Garcia-Bellido and Merriam 1971)] and *m* is the average clone size. Because generally only one of the daughter cells becomes *mwh*-labeled after the loss of the *mwh*^+^*Y* chromosome during mitosis, *m* needs to be multiplied by two. Screening *N* wings implies the analysis of *N C*/2*m* target cells exposed to the treatment and the number of these cells can easily reach 10^5^. Considering that a single *mwh*-labeled cell will give rise to one clone, *n*, the number of *mwh* clones in *N* wings, equals the number of the target cells that gave rise to daughter cells without the *mwh*^+^*Y* chromosome. Therefore *f*, the frequency of *mwh*^+^*Y* chromosome loss, is *f* = *n* 2*m*/*N C*.

To determine the spontaneous frequency of *mwh* clone formation, we analyzed wings of (1) *w*/*mwh*^+^*Y*; *mwh* males that developed (i) on standard *Drosophila* food or (ii) on the 4-24 instant *Drosophila* medium and (2) wings of *w*/*mwh*^+^*Y*; *mwh lds^hor-rvP2^*/*mwh lds*^+^ males. Because the frequency of the *mwh* clones were not significantly different in the aforementioned types of wings (86 clones/58 wings, 22/16 and 56/34, respectively; *P* > 0.05, χ^2^ test), we pooled the data and used the 164 *mwh* clones in 108 wings as the control frequency throughout the present study (Table 1).

The *mwh* clones were grouped into size classes. A size class defines the minimum number of cell divisions required between the induction of a clone and its formation to the observed size. The distribution of the *mwh* clones among the size classes seem to follow the “halve-by-half” rule, *i.e.*, there are twice as many clones in class I as in class II, twice as many in class II as in class III, and so on (Table 1). If we assume a (1) constant frequency of loss of the *mwh*^+^Y chromosome throughout the subsequent rounds of mitoses and (2) equal contribution of the wing disc cells to the final wing blade cell population, the 164 *mwh* clones are expected to be distributed as follows among size classes I-V: 85, 42, 21, 11, and 5. Because the observed (96, 39, 22, 5, and 2) and the expected distributions are not significantly different (*P* > 0.05, χ^2^ test), the incidence of *mwh*^+^*Y* chromosome loss appears to be constant throughout the subsequent rounds of cell divisions in cells of the wing discs (Table 1). The spontaneous frequency of *mwh*^+^Y chromosome loss is 1.7 × 10^−4^/cell division (Table 1). The spontaneous loss of the *mwh*^+^*Y* chromosome appears to happen randomly because the distribution of the 164 *mwh* clones among the 108 wings follows the Poisson distribution: the observed and the expected values are not significantly different (Table 2; *P* > 0.05; χ^2^ test). It may thus be safe to conclude that at least the spontaneous loss of the *mwh*^+^Y chromosome occurs randomly.

**Table 2:** Distribution of the spontaneous *mwh* clones in the control wings

	Number of wings with *N_i_ mwh* Clones						
	0	1	2	3	4	5	6
Observed	30	28	29	10	7	4	0
Calculated*^a^*	23.7	35.9	27.3	13.8	5.2	1.6	0.4

a Based on the Poisson distribution *P(i)*= ν*^i^* e*^–ν^*/*i!*, where *ν*=*n*/*N* and *n* =164, *N* = 108.

In principle, some of the *mwh* clones might have originated through position-effect-variegation (PEV) of the *mwh*^+^ transgene inserted into the *mwh*^+^*Y* chromosome. To test this possibility, we generated *w*/*mwh*^+^*Y* males that were homozygous for *mwh* and carried either *Su-var(2)1^03^*, a dominant suppressor mutation of PEV or *Su(var)3-9^ptn^*, an exceptionally strong PEV enhancer mutation (Schotta *et al.* 2003; Ebert *et al.* 2004). The number and size of the *mwh* clones were statistically not different form the control. Hence, PEV does not seem to play a role in the origin of the *mwh* mosaic spots.

### Features of *mwh* mosaicism induced by X-rays, colchicine, EMS, and formaldehyde

After the exposure of *w*/*mwh*^+^Y; *mwh* larvae to a 1000 Rad of X-rays, the frequency of the *mwh* clones greatly exceeded the control level (Table 1) in agreement with the fact that X-rays induce chromosome loss (Szabad and Wurgler 1987; Sgura *et al.* 2001). Although the irradiated larvae were 84- to 92-hr old, about three rounds of mitoses away from the cessation of cell proliferation, about 10% (17/119) of the *mwh* clones grew into size classes V-VIII clones (Table 1*)*. The formation of such relatively large clones is unusual. The simplest explanation is that excess cell division of the *mwh* homozygous cells is induced as the consequence of X-ray−induced cell death and intercalary regeneration (Haynie and Bryant 1977). As estimated by Haynie and Bryant (1977), 1000 Rad of X-rays reduces the number of cells capable of making a normal contribution to the adult wing by 40–60%. They also suggested that the effect was the consequence of radiation-induced aneuploidy. Extra rounds of cell divisions, in which the *mwh*-labeled cells seem to participate, replace the lost cells and hence larger than normal clones develop. The average clone size was 2.6 *mwh* cells and thus the frequency of *mwh*^+^*Y* chromosome loss is 17.2 × 10^−4^, about 10-fold the control level (Table 1).

Some of the X-ray−induced *mwh* clones on the wings of the *w*/*mwh*^+^Y; *mwh* males might have originated through the loss of function of the *mwh*^+^ gene in the *mwh*^+^*Y* chromosome. To estimate the contribution of the lost *mwh*^+^ gene function in the frequency of the *mwh* mosaic spots, we inserted the *mwh^+^* gene contained within the FlyFos-030330 clone (Ejsmont *et al.* 2009) into the *attP* docking site at 47C (*VK16*) on the right arm of the second chromosome (Venken *et al.* 2006, 2009). After X-irradiation at a 1000 Rad of *w*/*Y*; *VK16 mwh*^+^/*In(2LR)Gla*; *mwh* larvae, seven *mwh* clones developed on 40 wings (Table 1). A comparison of the 119/12 and the 7/40 frequencies clearly shows that the vast majority of the *mwh* clones on wings of the X-irradiated *w*/*mwh*^+^*Y*; *mwh* males originated due to chromosome loss and that the contribution of point mutations in the *mwh*^+^ gene is very low. It is also highly unlikely that the seven *mwh* clones (of 40 wings) originated through X-ray−induced mitotic recombination since the *In(2LR)Gla* chromosome effectively suppresses recombination in the 47C area where the *VK16* landing site is (Venken *et al.* 2006).

Colchicine binds tubulin and inhibits microtubule polymerization. Hence, colchicine effectively functions as a “mitotic poison” or spindle poison. It is therefore expected to induce a high frequency of *mwh* clones in wings of the *w*/*mwh*^+^*Y*; *mwh* males, as indeed shown in Table 1. The average size of the *mwh* clones was 2.8 cells after colchicine treatment, and the frequency of clone induction was 29.4 × 10^−4^. Although approximately 80% of the clones (50/63) appeared in the expected I-IV size classes, several grew unusually large and were assigned to classes V and VI (Table 1). This indicates that colchicine induces cell death in the wing primordia followed by intercalary regeneration, in agreement with previous data documenting that cells die most likely through the induction of aneuploidy (Isaenko *et al.* 2002).

EMS is routinely used as a mutagen in *Drosophila* (Lewis and Bacher 1968). It induces mostly point mutations and some chromosomal breaks in wing imaginal disc cells but does not appear to induce detectable levels of aneuploidy in germline cells (Szabad 1986). The present assay clearly shows that EMS induces the formation of *mwh* clones, although with low but significantly greater frequency as in the control (Table 1). We surmise that most of these clones are probably due to EMS-induced mutations in the *mwh^+^* gene present on the *mwh*^+^*Y* chromosome and to chromosomal loss. Indeed, a 4-hr 8 mM EMS treatment induced mutations at a rate of 8.8 × 10^−4^ in the wing disc cells, a value similar with the 4.6 × 10^−4^ value reported here (Table 1) (Szabad and Bennettova 1986).

Formaldehyde induced a subtle but significant elevation in the frequency of the *mwh* clones (Table 1). However, in line with the mosaic spots that originated through formaldehyde-induced chromosome breaks (Szabad *et al.* 1983), the *mwh* clones remained very small, and consequently the frequency of *mwh* clone formation was rather low: 2.8 × 10^−4^ (Table 1). The generally small size of the 136 *mwh* clones is most likely the consequence of a delay in action of the formaldehyde between its uptake in the digestive system and its ability to reach the wing disc cells. Formaldehyde has been known to induce mutations through small-scale chromosomal rearrangements without compelling evidence of induced chromosome loss in yeast and cultured mammalian cells (Zimmermann and Mohr 1992; Speit and Merk 2002; Speit *et al.* 2011). To elaborate on the origin of the *mwh* clones in wings of the *w/mwh*^+^*Y*; *mwh* males after formaldehyde treatment, we analyzed 20 wings of *w*/Y; *VK16 mwh*^+^/*In(2LR)Gla*; *mwh* males in which the *mwh* clones cannot be caused by chromosome loss or recombination. Because only two *mwh* clones formed (each with one *mwh* homozygous cell) on 20 such wings, this result is suggestive that at least some of the 136 *mwh* clones emerged due to formaldehyde-induced loss of the *mwh*^+^*Y* chromosome.

### Chromosome stability and *lodestar* gene function

The *lds^Hor-D^* mutation has been shown to induce chromosome instability and loss of chromosomes (Szabad *et al.* 1995; Szalontai *et al.* 2009). Molecular analysis revealed that *lds^Hor-D^* is a dominant-negative *lds* mutant allele and that the encoded A777T protein causes chromosome instability and loss (Szalontai *et al.* 2009). To assess the effect of *lds^Hor-D^* on instability of the *mwh*^+^Y chromosome, we generated *w*/*mwh*^+^*Y*; *mwh lds^Hor-D^*/*mwh lds*^+^ males and screened their wings for *mwh* mosaic spots. As shown in Table 1, the frequency of the *mwh* clones significantly exceeded the control value in the presence of *lds^Hor-D^* and the frequency of clone induction increased more than 3-fold, from 1.7 × 10^−4^ to 5.9 × 10^−4^. Hence, *lds^Hor-D^* causes chromosome instability and loss, not only in the female and male germline and during early embryogenesis (Szabad *et al.* 1995; Szalontai *et al.* 2009) but also in the wing disc cells during mitoses.

Because the level of *lds* gene expression is rather low in the imaginal disc cells (J. Szabad data not shown), we achieved greater levels of expression of the *lds^Hor-D^* allele by constructing *w*/*mwh*^+^*Y*; *nub-Gal4*/*UAS-lds^Hor-D^*; *mwh* males in which *nub-Gal4*, a wing disc specific driver ensured expression of a *UAS-lds^Hor-D^* transgene. The frequency of the *mwh* clone induction increased to 15.1 × 10^−4^ despite the fact that these wing disc cells carried two normal *lds*^+^ gene copies in addition to the *lds^Hor-D^* mutation (Table 1). Hence, the A777T mutant protein efficiently induces loss of the *mwh*^+^*Y* chromosome.

To analyze the role of the Lds protein in maintenance of chromosome stability, we constructed *w*/*mwh*^+^*Y*; *mwh lds^hor-rvP2l^*/*mwh Df(3R)ED5218* males that did not carry a functional *lds* gene. Wings of these males carried a significantly greater frequency of *mwh* clones than the control (164/24 *vs.* 164/108; Table 1), confirming that the *lds* gene is required for the maintenance of genome stability (Szalontai *et al.* 2009). The frequency of *mwh* clone induction was very similar in wings of the *lds^Hor-D^*-carrying males and in those that did not carry functional *lds* gene (5.9 × 10^−4^ *vs.* 8.7 × 10^−4^; Table 1), providing additional evidence for the dominant-negative nature of *lds^Hor-D^*.

## Discussion

Spindle assembly checkpoint and mitotic catastrophe are cellular machineries that guard over chromosome/genome stability in the course of the subsequent cell divisions (Musacchio 2011; Vitale *et al.* 2011). Failed or disturbed functions of these surveillance mechanisms lead usually to cell death. However, some of the cells may escape the attention of the aforementioned mechanisms and survive. Many of these cells are aneuploid and may become the source of mental retardation, miscarriage, and cancer (Pellman 2007; Gordon *et al.* 2012; Holland and Cleveland 2012; Pfau and Amon 2012). The aforementioned well-established findings necessitate the elaboration of robust, reliable, and cheap aneuploidy test procedures.

The evolutionary conserved nature of the mechanisms involved in the aforementioned phenomena and processes (Lince-Faria *et al.* 2009) call for the use of model species to detect chromosome gain and/or loss. *Drosophila melanogaster* is an appropriate model species for the analysis of numerous basic biologic processes, including mutagenesis (Bellen *et al.* 2010). There have been a number of *Drosophila*-based aneuploidy test procedures developed to detect gain and/or loss of chromosomes both in the germline and in the soma (Szabad 1986; Szabad and Wurgler 1987; Rodriguez-Arnaiz *et al.* 1992; Szabad *et al.* 1995). However, most of these techniques detect aneuploidy in the female and/or in the male germ line, and there are two major difficulties associated with the germ-line based procedures: (1) a limited number of the germline cells and (2) the long time course between the induction and the detection of the aneuploidy (Szabad and Bennettova 1986; Szabad *et al.* 1995). Imaginal discs with ongoing rounds of mitoses and a large number of target cells are ideal “tools” to detect aneuploidy. A previous method based on loss of a *white^+^Y* choromosome in photoreceptors (Szabad and Wurgler 1987) was shown to work but has not been included into the battery of the so-called genetic toxicity testing procedures (Zeiger 2004) because (1) small eye clones go undetected and hence the sensitivity of the procedure is rather low, and (2) detection and characterization of the eye clones is relatively complicated and time consuming. These caveats are clearly not an issue in this assay.

Indeed, the *Drosophila* wing blades appear to be an ideal organ to analyze cellular events. The wings develop as a sack of diploid epithelial cells (discs) in which the successive rounds of cell cycles occur at about 10-hr intervals, the cell number grows exponentially, and mitoses cease soon after pupariation (Bryant and Levinson 1985; Dubatolova and Omelyanchuk 2004; Baker 2007; Neto-Silva *et al.* 2009). Of the approximately 50,000 wing disc cells, about 30,000 compose the wing blade, a chitinous structure that is flat, highly convenient to mount and analyze, and in which practically every cell forms a trichome (Figure 1). The large collection of trichome marker mutations (Garcia-Bellido and Dapena 1974) set the wing discs apart as appropriate “tools” to study cellular events, including mutagenesis. Szabad *et al.* (1983) proposed the so-called somatic mutation and recombination test to detect chromosome breaks—through the use of the *mwh* and the *flare* marker mutations—and point mutations induced in the *mwh*^+^ gene (Szabad *et al.* 1983; Surjan *et al.* 1985; de Andrade *et al.* 2004). Regrettably, loss of the X (first), the second, or the third chromosomes bring about cell death, and the absence of one of the fourth chromosomes significantly reduces viability of the wing disc cells, but loss of the *Y* chromosome has no impact on cell viability. After the loss of the *mwh*^+^*Y* chromosome, the wing disc cells survive and propagate their new genetic composition to their descending cells that remain together and form an *mwh* clone in the wing blade (Figure 1 and 2). As described in the present work, formation of the *mwh* clones in wings of the *w*/*mwh*^+^*Y*; *mwh* males is thus a reliable indicator of chromosome loss. We also show that variegation of the *mwh*^+^ transgene or point mutations in the transgene play little if any contribution to the formation of the *mwh* clones.

The number and size of the *mwh* clones allow a quantitative evaluation of the effectiveness of the environmental or genetic “treatments” to induce the loss of the *mwh*^+^*Y* chromosome (Szabad *et al.* 1983). Our data show that chromosomal loss can be induced by X-rays, colchicine, and formaldehyde, whereas EMS does not cause chromosomal loss. Finally, gain- and loss-of-function mutations in *lodestar*, previously shown to induce chromosome instability, also cause chromosomal loss in our assay. In summary, the proposed assay is simple, sensitive and inexpensive.

Based on the present data, we propose the *w*/*mwh*^+^*Y*; *mwh* system is an adequate tool to detect *in vivo* the effects of environmentally and genetically induced chromosome loss in a higher eukaryotic organisms.
